# Chagas disease in Virgem da Lapa, Minas Gerais, Brazil: left ventricle aneurysm and the risk of death in the 24-year interval

**DOI:** 10.1590/0074-02760200056

**Published:** 2020-06-12

**Authors:** José Borges-Pereira, José Rodrigues Coura, Patrícia Lago Zauza, Claude Pirmez, Sérgio Salles Xavier

**Affiliations:** 1 Fundação Oswaldo Cruz-Fiocruz, Instituto Oswaldo Cruz, Laboratório de Doenças Parasitárias, Rio de Janeiro, RJ, Brasil; 2 Fundação Oswaldo Cruz-Fiocruz, Instituto Oswaldo Cruz, Rio de Janeiro, RJ, Brasil; 3 Fundação Oswaldo Cruz-Fiocruz, Instituto Nacional de Infectologia Evandro Chagas, Laboratório de Pesquisa Clínica em Doença de Chagas, Rio de Janeiro, RJ, Brasil

**Keywords:** Chagas disease, left ventricle aneurysm, risk of death, Virgem da Lapa, MG

## Abstract

**BACKGROUND:**

Left ventricular aneurysm (LVA) is indicator of high morbidity in Chagas’ disease. A cross-sectional study performed identified LVA in 18.8% of the chronic chagasic patients (CCP).

**OBJECTIVE:**

Determine the risk of death of patients with chronic chagasic cardiopathy (CCC) and LVA in 24-year interval.

**MATERIAL AND METHODS:**

In 1995 a cohort of 298 CCP was evaluated by anamnesis, physical examination, EKG and ECHO and classified in groups: G0 = 86 without cardiopathy; G1 = 156 with cardiopathy without LVA and G2 = 56 with cardiopathy and LVA. 38 patients of G0 and G1 used benznidazole. Information about the deaths was obtained in the notary, death certificates, hospital records and family members.

**FINDINGS:**

Were registered 113 deaths (37.9%): 107 (35.9%) attributed to cardiopathy and 6 (2.0%) to other causes (p < 0.05). Amongst these 107 deaths, 10 (11.6%) occurred in G0; 49 (31.4%) occurred in G1 and 48 (85.7%) occurred in G2 (p < 0.05). The risk of death was 2.7 and 7.4 times significantly higher in G2, than in G1 and G0, respectively.

**CONCLUSION:**

Chronic chagasic patients with LVA and ejection fraction < 45% have a higher risk of death than those without.

Chagas disease, caused by the protozoan *Trypanosoma cruzi*, affects about 6 million people in Latin America. It represents an important public health problem due to the high cardiac morbidity and persistence of its social, economic and cultural constraints, despite the significant reduction in vector transmission in the last thirty years, especially in the countries of the Southern Cone.^1^ The higher prevalence of heart disease in the chronic Chagas population compared to the non-Chagas population is evidenced in sectional and longitudinal clinical-epidemiological studies conducted, in several endemic areas of Brazil.^2^ ^,^ ^3^ ^,^ ^4^ ^,^ ^5^ ^,^ ^6^ ^,^ ^7^ Clinical-epidemiological studies have indicated cardiomegaly and complex arrhythmias as the main markers of severity of chronic Chagas heart disease.^8^ ^,^ ^9^ ^,^ ^10^ With the insertion of echocardiography in the cardio functional evaluation of Chagas disease patients, left ventricle aneurysms, especially in the apical region, were added in the gallery of markers of severity of Chagas heart disease,^11^ with prevalence ranging from 8% to 18%,^12^ ^,^ ^13^ ^,^ ^14^ ^,^ ^15^ with high potential generator of severe arrhythmias and thromboembolism.^16^ ^,^ ^17^ ^,^ ^18^ ^,^ ^19^

The presence of left ventricle aneurysm (LVA), especially apical, with moderate to severe impairment of ventricular function associated with low fractions of ejections has been blamed for the significant reduction in life expectancy of patients with chronic Chagas disease, in outpatient and hospital cohort studies.^16^ ^,^ ^17^ ^,^ ^18^ However, the etiopathogenesis of these aneurysms is not yet clearly defined. There are hypotheses that try to hold myocarditis accountable, mechanical factor, left anterior divisional block, conduction disorder and parasympathetic cardiac dysautonomia, according to the different studies all these factors can contribute to the generation of left ventricle aneurysms.^11^ ^,^ ^20^

The influence of LVA on death of the chagasic population has been poorly studied in a systematised way, despite the great importance it represents, especially to strengthen therapeutic decision-making capable of controlling the evolutionary process of the disease and improving the quality of life of these patients. In this context, we proposed this work to evaluate the participation of LVA in the death of chronic Chagas patients studying deaths recorded in the period 1995-2019, in a cohort of 298 Chagas patients from Virgem da Lapa, Minas Gerais, Brazil, in the expectation of offering evidence aimed improvement of individual and collective health.

## MATERIALS AND METHODS

**Fig. 1:**
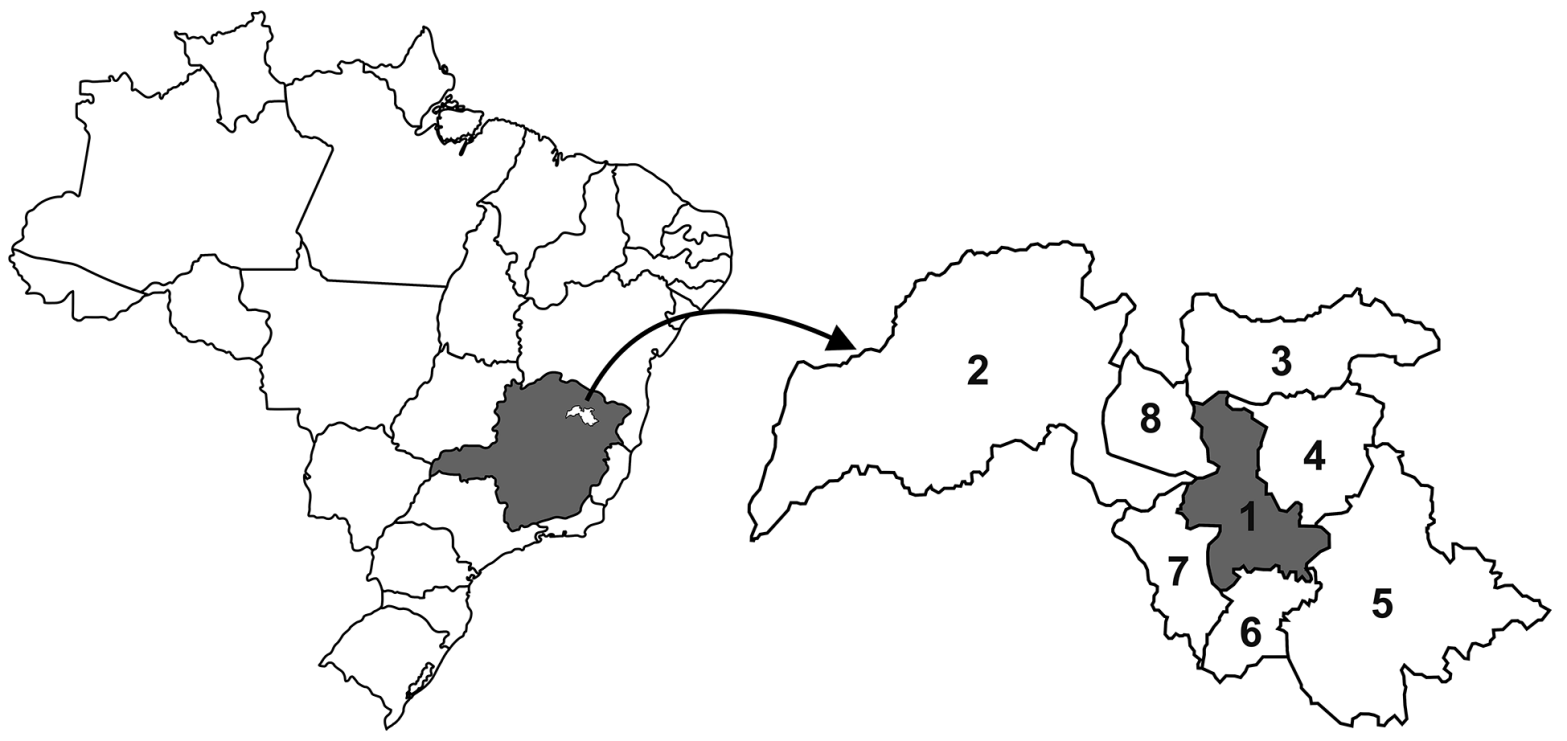
location of the study area (1- Virgem da Lapa) in the State of Minas Gerais, Brazil, and the municipalities bordering: 2- Grão Mogol; 3- Rubilita; 4- Coronel Murta; 5- Araçuaí; 6- Francisco Badaró; 7- Berilo; 8- Josenópolis.

*Area of study* - The study was conducted between March 1995 and September 2019, in the municipality of Virgem da Lapa, located in the middle Jequitinhonha Valley, State of Minas Gerais, Brazil (Fig. 1), with surface of 872 km^2^, altitude of 719 m, scarce vegetation and absence of native forest. The estimated population of 16,000 inhabitants^21^ with 60% residents in the urban area and 40% living in the rural area. In the period 1975-1980 prevalence of Chagas infection in the population of the rural area was estimated at 28.9%^22^ with a drop to 12.6% in the period 1976-1996, due to the effectiveness of vector control measures installed and maintained so far, it also configures the absence of Chagas infection in residents under the age of 30 years and the elimination of *Triatoma infestans* and *Panstrongylus megistus* from the households in the region.^22^

*Cohort* - It was composed of 298 seropositive individuals for anti-*T. cruzi* antibodies through recombinant ELISA and indirect immunofluorescence (IFI) tests,^23^ conducted in 1995 during a study on family morbidity. There are 184 (61,7%) women aged 17 to 90 years (mean = 51 ± 14 years) and 114 (38,3%) men aged 13 to 87 years (mean = 50 ± 14 years), showing greater proportion of women (p < 0.05), however, without significative difference in the average ages (p > 0.05). All were submitted to clinical examination, resting electrocardiogram (EKG) and two-dimensional echocardiogram (ECHO). According to the results of the tests performed in 1995, chronic chagasic cardiopathy (CCC = anormal EKG and /or ECO) was diagnosed in 212 (71.1%) patients, 87 (76.3%) among men and 125 (67.9%) among women (X^2^ = 2.40; p = 0.120); 88 (60.7%) between 145 patients aged ≤ 50 years and 124 (81.0%) between 153 patients over the age of 50 years (X^2^ = 15.02; p = 0.0001); 111 (72.6%) between 153 non-whites and 101 (69.7%) between 145 whites (X^2^ = 0.303; p = 0.581). Also based on the results of the exams, the patients were classified into three groups: **G0** = 86 patients without heart disease (normal EKG and ECHO); **G1** = 156 CCC carriers without LVA and **G2** = 56 CCC and LVA carriers. The demographic, clinical, electrocardiographic and echocardiographic variables studied are marked in Table I with emphasis on the higher frequency of extra-systoles and lower mean ejection fractions in the group of patients with LVA.

Of the 298 patients in the cohort, 286 had medical records in the health units of the municipality and 12 patients in group G0 were not registered. Continuous treatment for CCC mainly with the use of amiodarone, beta-blockers and angiotensin-converting enzyme inhibitors isolated or associated with the treatment of comorbidities such as hypertension, diabetes mellitus and dyslipidemias monitored by professionals of the basic health network of the municipality was identified in 105 (67.3%) patients in group G1 and 56 (100%) in G2 group. On the other hand, conventional etiological treatment with benznidazole (BZD) at different time intervals was performed in 23 patients in group G0 (without evident heart disease) and in 15 patients in group G1 (with mild heart disease).^1^

**TABLE I:** Characteristics of the groups of patients on 1995, Virgem da Lapa, Minas Gerais, Brazil

Characteristics	Group G0 (n = 86)		Group G1 (n = 156)		Group G2 (n = 56)		Statistical analysis G1 x G2	
	Total	%	Total	%	Total	%	X^2^ =	p < 0,05
Gender								
Female	59	68.6	96	61.5	29	51.8	1.61	No
Male	27	31.4	60	38.5	27	48.2		
Age (years)								
13-50	57	66.3	57	36.5	31	55.4	6.01	Yes
> 50	29	33.7	99	63.5	25	44.6		
Arterial hypertension								
Present	9	10.5	47	30.1	11	19.6	2.27	No
Absent	77	89.5	109	69.9	45	80.4		
Ventricular extrasystoles								
Present	0		49	31.4	32	57.1	11,5	Yes
Absent	86	100.0	107	68.6	24	42.9		
CBRB + LAH								
Present	0		61	39.1	22	39.3	0.006	No
Absent	86	100.0	95	60.9	34	60.7		
Ejection fraction < 45%								
Present	0		9	5.8	21	37.5	34.15	Yes
Absent	86	100.0	147	94.2	35	62.5		

G0: patients without cardiopathy; G1: patients with cardiopathy without left ventricle aneurysms; G2: patients with cardiopathy and left ventricle aneurysms; X^2^ = non-corrected chi-square; p < 0.05; Yes: significant statistical difference); no: non-statistically significant difference; CBRB + LAH: complete blockage of the right branch + left anterior hemiblock.

*Exams* - The clinical examination was shown to measure blood pressure in the right or left forearm with the sitting patient, anamnesis and physical examination directed to the cardiovascular system, according to standardisation of OMS/PAHO experts.^23^ Arterial hypertension (values ≥ 140 x 90 mm Hg) was defined according to the criteria of the Brazilian Society of Cardiology.^24^ The EKG was obtained with the registration of the twelve classical derivations, being at least three complexes by derivation and long D2 in case of arrhythmias; reading and interpreting were made by two experts, obeying the standards of the New York Heart Association (NYHA, 1973)^25^ considering normal frequencies from 60 to 120 bpm and sinus arrhythmias.

The ECHO was performed by a single researcher, co-author of this study, Phd Sérgio Salles Xavier, who was unaware of the clinical and electrocardiographic findings of patients. The ECHO obtaining images in conventional paraesternal, apical, supraesternal, subcostal and variations in order to identify segmental alterations of the type small aneurysms. The global systolic function of the left ventricle (LV) was evaluated at M mode by calculating the ejection fraction according to Teicholz et al.^26^ and also by subjective estimation from two-bidimensional echocardiography^27^ mainly due to the segmental pattern of myocardial involvement in the Chagas disease. The type of myocardial involvement was defined as segmental when in, at least, one segment the contractile deficit was significantly more pronounced than in the others, or diffuse when all segments had similar contractile deficit. In the segmental analysis, the model of 16 segments recommended by the American Society of Echocardiography^28^ was used and the segments were classified as normal, hypokinetic, akinetic and discinetic according to the criteria conventional.

*Deaths* - In September 2019, 24 years and 6 months after the initial examination, an active search was made for the 298 patients, defining residence, survival and death. Information on deaths was obtained and confirmed in notaries, death certificates, hospital records and among family members. Deaths were analysed in relation to the cause (cardiac and other causes), gender (women and men), age group (≤ 50 years and > 50 years), blood pressure (hypertensive and non-hypertensive), complete blockage of the right branch + left anterior hemiblock (CBRB + LAH present and absent) and ejection fraction (< 45% and ≥ 45%). Deaths from heart disease were considered in the following records: sudden death, death due to heart failure, death from Chagas disease, acute pulmonary edema, acute myocardial infarction and stroke in patients with cardiac arrhythmias and/or LVA; the other types of records were included in the group of other causes.

**Fig. 2:**
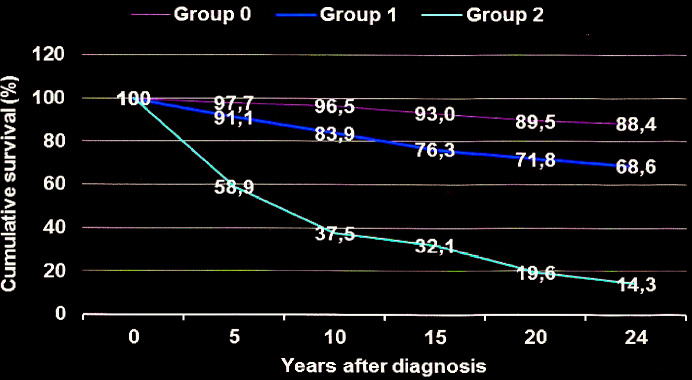
cumulative survival curves in groups of chronic chagasic patients: G0 - patients without cardiopathy; G1 - patients with cardiopathy without left ventricle aneurysms; G2 - patients with cardiopathy and left ventricle aneurysms. Statistical analysis: G2 vs G1; X^2^ = 48.96; p = 0,0000; RR = 2.72 (IC = 2.11 < RR< 3.52).

*Statistical and ethical analysis* - The data were submitted to statistical analysis through the package contained in the EPI-INFO.7 program, calculating the chi square of relative risk, considering the significance level for p ≤ 0.05. The Log-rank test was used to compare the survival curves of patient groups G0, G1 and G2 (Fig. 2). The project was approved by FIOCRUZ ethics committee, according to the recommendations of resolution 196/1996 of the Brazilian Ministry of Health, on ethics in research with human beings. All patients participated freely.

## RESULTS

*Initial cohort data* - Considering the results of the exams performed in the cohort of 298 chronic Chagas patients in 1995 (Table I), 86 (28.9%) did not show any evidence of heart disease (G0), while in 212 (71.1%) there was a diagnosis of heart disease (p < 0.05), 156 (52.4%) without aneurysms of the left ventricle and 56 (18.8%) aneurysms of the left ventricle (p < 0.05). Regarding gender, in the cohort, the proportion of women (184 = 61.7%) was significantly higher than that men (114 = 38.3%) (p < 0.05), a trend observed among patients in groups G0 and G1, but not among patients of group G2 (p > 0.05). Regarding the age of the patients, the initial mean of the cohort was 50 ± 14 years, while in the groups they were 44 ± 12 years in G0, 53 ± 14 years in G1 and 50 ± 14 years in G2. The analysis of variance of the mean G1 verse G2 indicated a non-significant different, however, both means were significantly higher than the initial mean of G0. Regarding age groups, in the cohort, 145 (48.7%) patients were ≤ 50 years of age (13 to 50 years) and 153 (51.3%) were ages above 50 years (51 to 90 years), a statistically non-significant difference (p > 0.05). In the analysis of each group, a higher percentage of patients aged ≤ 50 years in G0 was observed, a higher percentage of patients aged > 50 years in G1, while in group G2, there was no significant difference in the percentages of patients from both age groups.

Arterial hypertension was diagnosed in 67 (22.5%) patients, without difference statistically significantly among patients in groups G0, G1 and G2. Ventricular extrasystoles were recorded in 81 (27.2%) patients, with percentage significantly higher among patients in group G2. On the other hand, the association of complete blockage of the right branch with left anterior hemiblock (CBRB + LAH), recorded in 83 patients (27.8%), did not present a significant difference in prevalence among patients in groups G1 and G2.

The ejection fraction < 45% was diagnosed in 30 (10.1%) patients: 21 (37.5%) among the 56 patients in group G2 and nine (5.8%) among the 156 patients in group G1, a statistically significant difference, indicating an important association between LVA and low ejection fraction. Considering the frequencies of patients with LVA classified in group G2 compared with group G1, it is noted that there was no statistically significant difference in relation to gender, arterial hypertension and the presence of CBRB + LAH. However, was significantly higher the frequency of patients with age 13-50 years, with ventricular extra-systoles and with ejection fraction < 45% in this group (Table I).

*Death analysis* - In the 24-years interval of the study, 113 (37.9%) deaths were recorded in the cohort, 107 (35.9%) of which were attributed to heart disease and six (2.0%) other causes (RR = 17.8; p < 0.05). Emphasising that all 185 patients without death records had the state of life confirmed by our team and by the community health agents of Virgem da Lapa, highlighting that all are part of the cohort of 1,450 chagasic patients evaluated annually 44 years ago by the group laboratory of parasitic diseases of the Oswaldo Cruz Institute - Rio de Janeiro. Among the deaths attributed to heart disease, sudden death occurred six times more than death from congestive cardiac insufficiency (Table II). Noteworthy is the record of 10 deaths attributed to heart disease among patients in group G0, eight of them, 10 years after diagnosis, in patients with a history of arterial hypertension and irregular treatment and two associated with stroke and history of diabetes mellitus and hypertension. Stroke was blamed for 10 deaths (five patients with apical LVA, three with complex ventricular extra-systoles and two with hypertension and diabetes mellitus). Among the six deaths from other causes are septicemia (n = 2), esophagus cancer (n = 1), pneumonia (n = 1), liver cirrhosis (n = 1), and acute pancreatitis (n = 1).

**TABLE II:** Causes of deaths recorded in the cohort of chronic chagasic patients in the 24-year interval (1995-2019), Virgem da Lapa, Minas Gerais, Brazil

Causes of deaths recorded	Group G0	Group G1	Group G2	Total
To cardiopathy	10	49	48	107
Sudden death	2	19	18	39
Ignored	4	6	5	15
Acute myocardial infarction	0	3	2	5
Congestive heart failure	2	5	7	14
Acute lung edema	0	0	3	3
Chronic Chagas heart disease	0	9	6	15
Stroke	2	3	5	10
Chagas disease	0	3	1	4
Cardiorespiratory arrest	0	1	1	2
To other causes	2	4	0	6
Acute pancreatitis	0	1	0	1
Esophageal cancer	0	1	0	1
Septicemia	0	2	0	2
Pneumonia	1	0	0	1
Liver cirrhosis	1	0	0	1
TOTAL	12	53	48	113

G0: patients without cardiopathy; G1: patients with cardiopathy without left ventricle aneurysms; G2: patients with cardiopathy and left ventricle aneurysms.

Of the 107 deaths attributed to heart disease, 10 (11.6%) occurred among patients in group G0, 49 (31.4%) among patients in group G1 and 48 (85.7%) among patients in group G2. The analysis of these frequencies expressed in Table III showed a risk of death 2.7 times higher among patients in group G2 compared to patients in group G1 (p < 0.05) and 7.4 times higher than among patients in group G0 (p < 0.05). Emphasising that the mean age of patients who died in group G2 (50.6 ± 13 years) was significantly lower than the mean age of deaths in group G1 (61.5 ± 16 years) and in group G0 (58.5 ± 14 years).

**TABLE III:** Percentage of deaths to cardiopathy in groups of chronic Chagas patients of Virgem da Lapa-MG, Brazil, in the interval of 24 years (1995-2019)

Group of patients	Number	Death	% death
G0 - without cardiopathy	86	10	11.6
G1 - with cardiopathy without left ventricle aneurysms	156	49	31.4
G2 - with cardiopathy and left ventricle aneurysms	56	48	85.7
TOTAL	298	107	35.9

G2 vs G1: X^2^ = 48.96; p = 0.000; risk ratio = 2.72.

The mean time intervals between the initial diagnosis and the deaths of the patients, expressions of life expectations, were 13 ± 7 years in group G0, 10.6 ± 6.6 years in group G1 and 8 ± 6.4 years in group G2, statistically significant differences. Emphasising that 42% of patients in group G2 died in the first five years after the diagnosis of heart disease, indicating the low life expectancy of patients with LVA and ejection fraction < 45%.

**Fig. 3:**
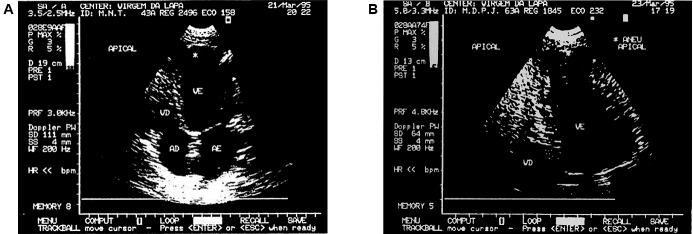
left ventricular apical aneurysm: (A) in the patient 2496, MNT, female, 43 years old, white, ejection fraction = 43%, electrocardiogram (EKG) with complete blockage of the right branch and ventricular extra-systoles, sudden death 98 months after diagnosis and (B) in the patient 1845, MDPJ, female, 63 years old, black, ejection fraction = 43%, EKG with complete blockage of the right branch, left anterior hemiblock and ventricular extra-systoles to pairs, sudden death 36 months after diagnosis.

Considering the percentages of deaths due to heart disease in patient groups at different intervals time between the initial diagnosis and deaths, the life expectancy curve was constructed (Fig. 2), in which it is observed that only 14.3% of the patients in group G2 were likely to be alive in the 24-years interval, while in groups G1 and G0 the odds were around 68,6% and 88,4%, respectively. Given these life expectations we can suggest a *good* prognosis for patients in group G0, *regular* for patients in group G1 and *bad* for patients in group G2. Fig. 3 presents apical aneurysm images of the left ventricle in patients of different ages, with moderate impairment of ventricular function and evolution for deaths in 98 and 36 months after diagnosis.

In a univariate analysis of Table IV data, it is observed that the frequency of deaths among patients in group G2 was significantly higher than among patients in group G1 regardless of gender, age group, hypertension, ventricular extra-systoles and complete blockage of the right branch associated with left anterior hemiblock. However, in relation to the ejection fraction < 45% there was no significant difference between the frequencies of deaths between patients in groups G1 and G2, showing the degree of independence of the ejection fraction < 45% in the production of the death outcome. Highlight for the data that all patients with apical LVA and ejection fraction < 45% died in the mean lifespan of four years.

**TABLE IV:** Percentage of death to cardiopathy on groups G1 and G2 of chronic chagasic patients, on interval 24 years (1995-2019), according with some variables, Virgem da Lapa, Minas Gerais, Brazil

Variables	Group G1 (n = 156)			Group G2 (n = 56)			Statistical analysis*	
	total	death*	% death	total	death*	% death	RR =	p < 0.05
Gender								
Female	96	31	32.3	29	25	86.6	2.7	Yes
Male	60	18	30.0	27	23	85.2	2.8	Yes
Statistical analysis p < 0.05			No			No		
Age (years)								
13-50	57	11	19.3	31	25	80.6	4.2	Yes
> 50	99	38	38.4	25	23	92.0	2.4	Yes
Statistical analysis p < 0.05			Yes			No		
Arterial Hypertension								
Present	47	20	42.6	11	9	81.8	5.5	Yes
Absent	109	29	26.6	45	39	86.7	3.3	Yes
Statistical analysis p < 0.05			Yes			No		
Ventricular extrasystoles								
Present	49	20	40.8	32	28	87.5	2.1	Yes
Absent	107	29	27.1	24	20	83.3	3.1	Yes
Statistical analysis p < 0.05			No			No		
CBRB + LAH								
Present	61	16	26.2	22	20	90.9	3.5	Yes
Absent	95	33	34.7	34	28	82.3	2.4	Yes
Statistical analysis p < 0.05			No			No		
Ejection fraction < 45%								
Present	9	8	88.9	21	21	100.0	1.1	No
Absent	147	41	27.9	35	27	77.1	2.7	Yes
Statistical analysis p < 0.05			Yes			Yes		

G1: patients with cardiopathy without left ventricle aneurysms; G2: patients with cardiopathy and left ventricle aneurysms; *: Epi info 7 (statcalc, non-corrected chi-square); RR: risk ratio; CBRB + LAH: complete blockage of the right branch + left anterior hemiblock.

In group G1, a death attributed to the CCC occurred in 27 (25.7%) of the 105 patients treated for heart disease and 22 (43.1%) of the 51 untreated (X^2^ = 4.83; p = 0.027; RR = 1.67), while in group G2 (all patients with LVA and treated for heart disease) death occurred in 48 (85.7%) of the 56 patients. These results indicate that adequate and continuous treatment of heart disease is capable of reducing mortality and offering better quality of life to chronic chagasic patients. The analysis of deaths among patients treated with BZD showed: (a) in group G0 there was no death among the treated patients (n = 23) and untreated (n = 63), (b) in group G1 there were 1 (6.7%) death among the 15 patients treated and 48 (34.0%) deaths among the 141 untreated patients (X^2^ = 4.71; p = 0.029; RR = 5.10), indicating etiological treatment as a reduction in mortality among patients with heart disease considered mild.

## DISCUSSION

In 1995 and 2007 our research group conducted sectional studies on the clinical-epidemiological importance of left ventricle aneurysms in populations with chronic infection by *T. cruzi*, respectively in the municipalities of Virgem da Lapa in Minas Gerais and João Costa and São João do Piauí, in Piauí-Brazil, in which significant regional differences in prevalence were identified. In Virgem da Lapa, the study considered a pioneer in the echocardiographic evaluation of a chronic chagasic population not selected in Brazilian rural areas, in the cohort of 298 chronic Chagas patients of both sexes and mean age of 50 ± 14 years were identified 56 (18,8%) aneurysms of the left ventricle, 38 (12.7%) in the apical region, with no significant difference in relation to gender, age, ethnicity and arterial hypertension, emphasising the presence of thrombi in the apical region in two patients, for whom platelet anti-aggregating medication and follow-up were prescribed. In both studies, a strong association was observed between left ventricle aneurysms with palpitations, ventricular extra-systoles, ejection fraction < 45%, with no difference regarding the compromised segment.^14^ In the longitudinal line of investigation on the impact of ventricular aneurysms on patients with chronic Chagas disease, Xavier^31^ in 1999 in a doctoral thesis diagnosed 96 (15.9%) aneurysms of the left ventricle, 89 (14.7%) in the apical region with varying sizes, with no difference in gender, with an important proportion of patients with moderate to severe impairment of ventricular function in an urban cohort of 604 patients of both sexes, mean age of 46.6 ± 12 years, being the natural majority of Bahia and Minas Gerais and coming from serological screening performed in blood banks, for this reason guarding close residents of the city of Rio de Janeiro, similar to the clinical-epidemiological profile of cohorts established in Virgem da Lapa and in the Sertão do Piauí. In 2005 Xavier et al.^16^ diagnosed 140 (13.3%) patients with apical aneurysm, of which 6% with mural thrombi in an urban cohort of 1,053 patients with chronic Chagas disease.

These studies involving cohorts with hundreds of patients, as well as studies with small cases of hospital environments^12^ ^,^ ^13^ ^,^ ^14^ ^,^ ^15^ ^,^ ^16^ ^,^ ^20^ format in their conclusions: (a) higher prevalence of left ventricle aneurysms in patients with chronic Chagas heart disease, compared to other myocardiopathies; (b) occurrence of LVA in all age groups, without statistically significant difference, behavior differing from the prevalence curve of chronic Chagas heart disease evaluated by electrocardiogram and megaesophagus identified by radiographic examination^30^ ^,^ ^31^ that significantly increase with the age of patients, indicating that the prevalence of LVA is not significantly associated with the time-of-disease evolution factor, probably phenomena intercurrent etiopathogenic stemming from tissue parasitic multiplication; (c) greater severity of the clinical picture of chagasic patients with ventricle aneurysms. Among the hypotheses, the proposal by Andrade^11^ stands out that “*apical aneurysm, highly characteristic of Chagas heart disease, may be associated with the impairment of the conduction system by creating inactivated areas or areas where the electrical stimulus would arrive late, which would be more susceptible to effects of intraventricular pressure during systole.”* Most studies on this theme do not confirm a significant association between tip aneurysm and His or auriculus-ventricular beam blocks and arterial hypertension.^16^ ^,^ ^22^ ^,^ ^30^ Still in the field of hypotheses, although not the object of investigation in the present study, we admit participation of the activations of parasite niches producing injuries and release of parasites for blood circulation thus justifying the greatest persistence patent parasitaemia among patients in group G2 (with left ventricle aneurysms) compared to patients in group G1 (without aneurysms), to be able to study systematised.

Evaluating the epidemiological and clinical aspects of the cohort of the present study, we observed that the frequency of LVA at the time of diagnosis was not statistically significant associated with gender, age group, arterial hypertension and complete blockage of the right branch associated with left anterior hemi-blockage. However, the presence of ventricular extra-systoles and the ejection fraction < 45% were significantly associated, consigning the close association of LVA with cardiac arrhythmias and impaired ventricular function, according to the literature reports.^13^ ^,^ ^14^ ^,^ ^15^ ^,^ ^31^ The highest frequency of death among patients in group G2 compared to patients in group G1 was not associated with gender, age, arterial hypertension, complete blockage of the right branch associated with left anterior hemi-blockage and ventricular extra-systoles configuring the force which LVA presented itself in the determinism of death regardless of these variables. However, as revealed by the results of outpatient, hospital and present cohort cases, LVA is a factor of severity, determinant of low life expectancy of chronic Chagas patients. Despite the relevance, the literature does not record a cohort longitudinal study older than two decades of duration, similar to that present, in which the mortality of chronic Chagas patients with left ventricle aneurysms was compared with chronic Chagas patients who do not have these lesions, natural from the same endemic area. However, we found sectional studies on chronic Chagas heart disease that configure LVA as a marker of severity of the clinical evolution of Chagas disease, configured at high prevalence, low function ventricular, high frequency of complex ventricular extra-systoles and stroke, inducers of lower life expectancy of patients and poor prognosis. In the cohort of the present study the severity of LVA can be formatted by the risk of death 2.7 times higher among patients in group G2 compared to patients in group G1, for the shorter mean life span and the higher occurrence of stroke among patients in group G2 compared to the results in group G1.

In the panel of causes of deaths in the cohort under study, similar to the reports of the literature, sudden death predominates over death due to congestive heart failure, usually in hospital, with insidious installation. It is noteworthy that deaths attributed to heart disease in group G0 are in accordance with longitudinal studies that reveal a good prognosis of patients in the indeterminate form for at least 10 years after diagnosis.^9^ ^,^ ^10^ ^,^ ^22^

The record of higher frequency of deaths due to stroke among chronic Chagas patients with LVA in the current cohort confirms the results in the literature, which has been attributed to the high thromboembolic potential of this lesion.^17^ ^,^ ^18^ ^,^ ^19^ ^,^ ^29^ ^,^ ^30^ In view of this outcome, Sousa et al.^18^ evaluated the importance of the use of anticoagulants in the prevention of ischemic stroke among the CCP concluding the benefit of the prescription in the clinical evolution of these patients and Carod-Artal et al.^30^ in a control group study evaluated risk factors for ischemia in 94 CCP treated at the Sara Brasília hospital, concluding that Chagas cardiomyopathy is independently associated with ischemic stroke and that the significant variables that predicted stroke patients in a stepwise logistic regression model were apical aneurysm, heart failure, arrhythmia to EKG, women and arterial hypertension, emphasising that the last two variables presented predominant bias in the study group.

Although the aim of this study was not to evaluate the influence of CCC treatment and etiological treatment of *T. cruzi* infection with BZD on mortality in this cohort, there was a lower risk of death among patients undergoing CCC treatment, as well as among patients treated with BZD, confirming data from the literature showing lower incidence and progression of CCC in the group of patients treated with BZD, reflecting on the reduction of mortality as well as in the better quality of life of patients monitoring the treatment of CCC.^32^ ^,^ ^33^ ^,^ ^34^

In short, in the 24-year evaluation interval, heart disease was considered responsible for 11.6% of deaths among patients without heart disease (G0), in 31.4% of deaths among patients with heart disease without left ventricle aneurysms (G1) and in 87.5% of deaths in patients with heart disease and left ventricle aneurysms (G2), translating as a good prognostic for patients in group G0, regular prognostic for patients in group G1 and in bad prognostic for patients in group G2 indicating the need for investigations on treatments capable of reducing the severity of chronic Chagas disease.

## References

[1] Dias JCP, Ramos NA, Gontijo ED, Luquetti A, Shikanai-Yasuda MA, Xavier SS (2016). II Brazilian Consensus on Chagas Disease, 2015.. Epidemiol Serv Saude.

[2] Chagas C, Villela E. (1922). Cardiac form of American Trypanosomiasis.. Mem Inst Oswaldo Cruz.

[3] Coura JR, Abreu LL, Dubois LEG, Correia-Lima F, Arruda E, Willcox HPF (1984). Morbidade da doença de Chagas. II - Estudos seccionais em quatro áreas de campo no Brasil.. Mem Inst Oswaldo Cruz.

[4] Borges-Pereira J, Willcox HP, Coura JR (1985). Morbidade da doença de Chagas. III - Estudo longitudinal de seis anos, em Virgem da Lapa, MG, Brasil.. Mem Inst Oswaldo Cruz.

[5] Borges-Pereira J, Zauza PL, Galhardo MC, Nogueira JS, Pereira GROL, Cunha RV. (2001). Chagas disease in the urban population of the sanitary district of Rio Verde, Mato Grosso do Sul, Brazil. Rev Soc Bras Med Trop.

[6] Borges-Pereira J, Castro JAF, Campo JHF, Nogueira JS, Zauza PL, Marques P (2002). Study of the infection and morbidity of Chagas’ disease in municipality of João Costa - National Park Serra da Capivara, Piauí, Brazil.. Rev Soc Bras Med Trop.

[7] Mott E, Lehman JS, Hoff R, Morrow RH, Muniz TM, Sherlock I (1976). The epidemiology and household distribution of seroreactivity to Trypanosoma cruzi in a rural community in Northeast Brazil.. Am J Trop Med Hyg.

[8] Laranja FS, Dias E, Nobrega GC, Miranda A. (1956). Chagas disease: a clinical epidemiological and pathologic study. Circulation.

[9] Kloetzel K, Dias JCP (1968). Mortality in Chagas disease: life table for the period 1949-1967 in an unselected population. Rev Inst Med Trop São Paulo.

[10] Dias JCP, Kloetzel K. (1968). The prognostic value of the electrocardiographic features of chronic Chagas disease. Rev Inst Med Trop São Paulo.

[11] Andrade ZA (1956). Apical lesion of the heart in chagasic chronic myocarditis. Hospital.

[12] Acquatella H, Schiller NB, Puigbó JJ, Giordano H, Suarez JA, Casal H (1980). M-mode and two-dimensional echocardiography in chronic Chagas’ heart disease. Circulation.

[13] Borges-Pereira J, Xavier SS, Pirmez C, Coura JR. (1998). Chagas’ disease in Virgem da Lapa County, Minas Gerais State, Brazil. IV. Clinical and epidemiological aspects of the left ventricle aneurysm.. Rev Soc Bras Med Trop.

[14] Viotti RJ, Vigliano C, Loucella S, Lococo B, Petti M, Bertocchi G (2003). Value of echocardiography for diagnosis and prognosis of chronic Chagas disease cardiomyopathy without heart failure. Heart.

[15] Borges-Pereira J, Xavier SS, Sousa AS, Castro JAF, Zauza PL, Coura JR (2007). Prevalence of left ventricular aneurysms among chronic Chagas disease patients from two areas in the State of Piauí, Brazil. Rev Soc Bras Med Trop..

[16] Xavier SS, de Sousa AS, do Brasil PEAA, Gabriel FG, de Holanda MT, Hasslocher-Moreno A (2005). Apical aneurysm in the chronic phase of Chagas disease: prevalence and prognostic value in an urban cohort of 1053 patients.. Rev SOCERJ.

[17] Albanesi-Filho FM, Gomes JB. (1991). Thromboembolism in patients with apical injury caused by chronic Chagas heart disease. Rev Port Cardiol.

[18] Sousa AS, Xavier SS, Freitas GR, Moreno AH (2008). Strategies for preventing cardioembolic stroke in Chagas disease. Arq Bras Cardiol.

[19] Barbosa-Ferreira JM, Nobre AF, Maldonado JGA, Borges-Pereira J, Zauza PL, Coura JR (2010). Ischemic stroke in a chronic chagasic patient autochthonous from the Brazilian Amazon.. Rev Soc Bras Med Trop.

[20] Carvalhal S, Bittencourt LAK, Nogueira EA. (1980). Apical lesion in Chagas heart disease. Arq Bras Cardiol.

[21] Camargo ME, Silva JR, Castilho EA, Silveira AC. (1984). Serological survey of the prevalence of chagasic infection in Brazil, 1975-1980. Rev Inst Med Trop São Paulo.

[22] Borges-Pereira J. (1997). Human Chagas disease: study of chronic infection, morbidity and mortality in Virgem da Lapa, Minas Gerais (1976-1996). [PhD Thesis in Tropical Medicine]. Rio de Janeiro:. Fundação Oswaldo Cruz.

[23] OMS, OPAS (1974). Aspectos clínicos de la enfermedad de Chagas. Informe de una reunión conjunta OMS/OPAS de investigadores. Bol Ofic Santaria Panamericana.

[24] SBC (2016). 7ª Diretriz Brasileira de Hipertensão arterial. SBC.

[25] NYHA (1973). Nomenclature and criteria for diagnosis of disease of the heart and great vessels.. Little and Brow Company.

[26] Teicholz LE, Kreuler T, Werman MV (1976). Problems in echocardiographic volume determinations: echocardiographic-angiocardiographic correlations in the presence or absence of asynergy. Am J Cardiol.

[27] Amico AF, Lichtenberg GS, Reisner SA, Stone CK, Schwartz RG, Meltzer RS (1989). Superiority of visual versus computerized echocardiographic estimation of radionuclide left ventricular ejection fraction. Am Heart Journal.

[28] Schiller NB, Shah PM, Crawtord M, DeMana A, Devereux R, Feigenbaum H (1989). Recommendations for quantitation of the left ventricle by two-dimensional echocardiography. J Am Soc Echocard.

[29] Taleb MM. (2017). An infrequent cause of apical ventricular aneurysm in the United States.. Gulf Heart Association.

[30] Carod-Artal FJ, Vargas AP, Horan TA, Nunes LGN (2005). Chagasic cardiomyopathy is independently associated with ischemic stroke in Chagas disease.. Stroke.

[31] Xavier SS (1999). Longitudinal study of cardiac morbidity and mortality of Chagas disease in a cohort of a large urban center: clinical, electrocardiographic, radiological and echocardiographic analysis of 604 cases. [PhD Thesis in Medicine-Cardiology].. Universidade Federal do Rio de Janeiro.

[32] Cançado JR (2002). Long term evaluation of etiological treatment of Chagas disease with benznidazole.. Rev Inst Med Trop São Paulo.

[33] Lana M, Lopes LA, Martins HR, Bahia MT, Machado-de-Assis GF, Wendling AP (2009). Clinical and laboratory status of patients with chronic Chagas disease living in a vector-controlled area in Minas Gerais, Brazil, before and nine years after aetiological treatment. Mem Inst Oswaldo Cruz.

[34] Zauza PL (2016). Impact of repetitive treatment with benznidazol in humoral immune response, parasitaemia and cardiopathy in chronic chagasic patients of Virgem da Lapa, Vale do Jequitinhonha, Minas Gerais. [PhD Thesis in Tropical Medicine].. Fundação Oswaldo Cruz.

